# Suboptimal antimicrobial discharge prescriptions at a tertiary referral children’s hospital

**DOI:** 10.1017/ash.2023.488

**Published:** 2023-12-04

**Authors:** Yi Wolf Zhang, Sruti Paturi, Lauren M. Puckett, David Scheinker, Hayden T. Schwenk, Torsten A. Joerger

**Affiliations:** 1 Department of Pediatrics, School of Medicine, Stanford University, Stanford, CA, USA; 2 Department of Management Science and Engineering, School of Engineering, Stanford University, Stanford, CA, USA; 3 Department of Pharmacy, Lucile Packard Children’s Hospital Stanford, Stanford, CA, USA; 4 Clinical Excellence Research Center, School of Medicine, Stanford University, Stanford, CA, USA; 5 Department of Pediatrics, Division of Infectious Diseases, Stanford School of Medicine, Stanford, CA, USA; 6 Department of Pediatrics, Division of Infectious Diseases, Children’s Hospital of Philadelphia and University of Pennsylvania Perelman School of Medicine, Philadelphia, PA, USA; 7 Formerly Department of Pediatrics, Division of Infectious Diseases, Stanford School of Medicine, Stanford, CA, USA

## Abstract

**Objective:**

To determine the rate of and factors associated with suboptimal discharge antimicrobial prescribing at a tertiary referral children’s hospital.

**Design:**

Retrospective cohort.

**Setting:**

Tertiary referral children’s hospital.

**Population:**

All enteral antimicrobial discharge prescriptions at Lucile Packard Children’s Hospital Stanford from January 1st, 2021 through December 31st, 2021.

**Method:**

All enteral discharge antimicrobials are routinely evaluated by our antimicrobial stewardship program within 48 hours of hospital discharge. Antimicrobials are determined to be optimal or suboptimal by an antimicrobial stewardship pharmacist after evaluating the prescribed choice of antimicrobial, dose, duration, dosing frequency, and formulation. The rate and factors associated with suboptimal antimicrobial discharge prescribing were evaluated.

**Results:**

Of 2,593 antimicrobial prescriptions ordered at discharge, 19.7% were suboptimal. Suboptimal prescriptions were due to incorrect duration (72.2%), dose (31.0%), dose frequency (23.3%), drug choice (6.5%), or formulation (5.7%). In total, 87.2% of antimicrobials for perioperative prophylaxis and 13.5% of treatment antimicrobials were suboptimal. Antimicrobials with the highest rate of suboptimal prescriptions were amoxicillin-clavulanate (40.7%), clindamycin (36.6%), and cephalexin (36.6%).

**Conclusion:**

Suboptimal antimicrobial discharge prescriptions are common and present an opportunity for antimicrobial stewardship programs during hospital transition of care. Factors associated with suboptimal prescriptions differ by antimicrobial and prescribed indication, indicating that multiple stewardship interventions may be needed to improve prescribing.

## Introduction

Antimicrobial stewardship programs (ASPs) consist of physicians, pharmacists, and other healthcare providers whose goal is to optimize antimicrobial prescribing. A prescription may be suboptimal if it deviates from evidence-based or best-practice recommendations, with regard to choice of antimicrobial, formulation, dose, or dosing frequency. Antimicrobial stewardship programs work to optimize antimicrobial prescribing and have been shown to decrease antimicrobial use and resistance, reduce cost, decrease adverse events associated with medications, and improve patient outcomes.^1–4^ The important role ASPs play in public health is further highlighted by the fact that these programs are an accreditation requirement of the Joint Commission.^5^ Pediatric ASPs face a number of additional challenges compared to their adult counterparts, including a smaller antimicrobial stewardship evidence base, need for weight-based dosing, and challenging patient populations, including premature neonates.^2^ In addition, growing and developing children pose dosing challenges, including variable pharmacokinetic and pharmacodynamic properties by age and dosing inferred from studies done in adults.^2^ Studies have shown children’s hospitals with ASPs are more effective in reducing antibiotic usage than hospitals without ASPs.^6^

Antimicrobial stewardship program efforts have typically focused on inpatient activities, but a growing body of literature highlights the importance of stewardship activities at transitions of care, including hospital discharge.^7–9^ Roughly one-third of hospitalized children are prescribed an antibiotic during their admission, and it is likely that many of these children are discharged on an antimicrobial.^10^ To date, there is a dearth of literature examining the appropriateness of pediatric hospital discharge antimicrobial prescribing.^11,12^ The aim of this study was to determine the rate of suboptimal antimicrobial discharge prescribing in a tertiary referral children’s hospital and to identify factors associated with suboptimal prescribing.

## Methods

### Study design and setting

This was a single-center, retrospective cohort study of antimicrobial discharge prescriptions at Lucile Packard Children’s Hospital Stanford (LPCHS) from January 1st through December 31st, 2021. Lucile Packard Children’s Hospital Stanford is a 361-bed tertiary referral children’s hospital with robust surgical, oncologic, and transplant populations as well as dedicated neonatal, pediatric, and cardiac intensive care units. The ASP at LPCHS monitors both inpatient and outpatient antimicrobial prescribing to promote the appropriate use of antimicrobials and reduce antimicrobial resistance and the spread of infection caused by multi-drug-resistant organisms.

### Study population and auditing process

Starting in November 2020, a single ASP pharmacist began daily audits of all enteral antimicrobial prescriptions sent at hospital discharge. Prescriptions sent Monday through Thursday were reviewed within 24 hours, while those ordered Friday through Sunday were reviewed the following Monday (i.e., within 72 hours). Prescriptions sent after the daily audit was conducted were included in the review on the next business day. Inhaled, topical, and injectable medications are not audited. All prescriptions were determined to be optimal or suboptimal based on the choice of antimicrobial, antimicrobial dose, formulation, dosing frequency, and duration. For prescriptions identified as suboptimal, the ASP pharmacist assessed whether the current prescription was a risk to patient safety (e.g., decreased efficacy, toxicity) and evaluated for opportunities for antimicrobial optimization. If patient safety was assessed to be compromised, the ASP pharmacist discussed recommendations with the prescriber for a revised prescription. Discharge prescription audits were recorded in a secure Excel database to identify trends and areas of opportunity for stewardship interventions. Antimicrobial stewardship program documentation included audit date, prescribing service, antimicrobial indication, and audit result. This database served as the source of our study population. All audits for enteral antimicrobial discharge prescriptions written for an infectious indication were included in the analysis.

### Variables and outcomes

For each antimicrobial prescription, the primary outcome was the classification of optimal or suboptimal. A prescription was classified as optimal based on alignment with national and/or institutional guidelines and based on the clinical judgment of the auditing ASP pharmacist.^13,14^ The following prescription issues were documented if determined to be the reason an antimicrobial prescription was suboptimal: 1) incorrect choice of drug (e.g., bug-drug mismatch); 2) inappropriate duration; 3) incorrect dose; 4) formulation dose mismatch (e.g., prescription of “high-dose” amoxicillin-clavulanate using the 7:1 formulation of amoxicillin-clavulanate); or 5) incorrect dosing frequency. Of note, a single suboptimal prescription may have had multiple documented issues. Additional patient-level information was extracted from the electronic health record (EPIC, Epic Systems Inc), including the patient’s sex, age, insurance type (public or private), and race/ethnicity. Prescriptions were further grouped based on the prescribed indication: treatment, medical prophylaxis, or perioperative prophylaxis. Secondary outcomes include the reason for suboptimal classification and the frequency of suboptimal prescribing by medical service, prescription indication, and antimicrobial type.

### Statistical analysis

All extracted data were uploaded and analyzed in Tableau Desktop 2021.4. Descriptive statistics, including frequencies for categorical variables and medians with interquartile ranges for continuous variables, were calculated.

## Results

**Figure 1.**
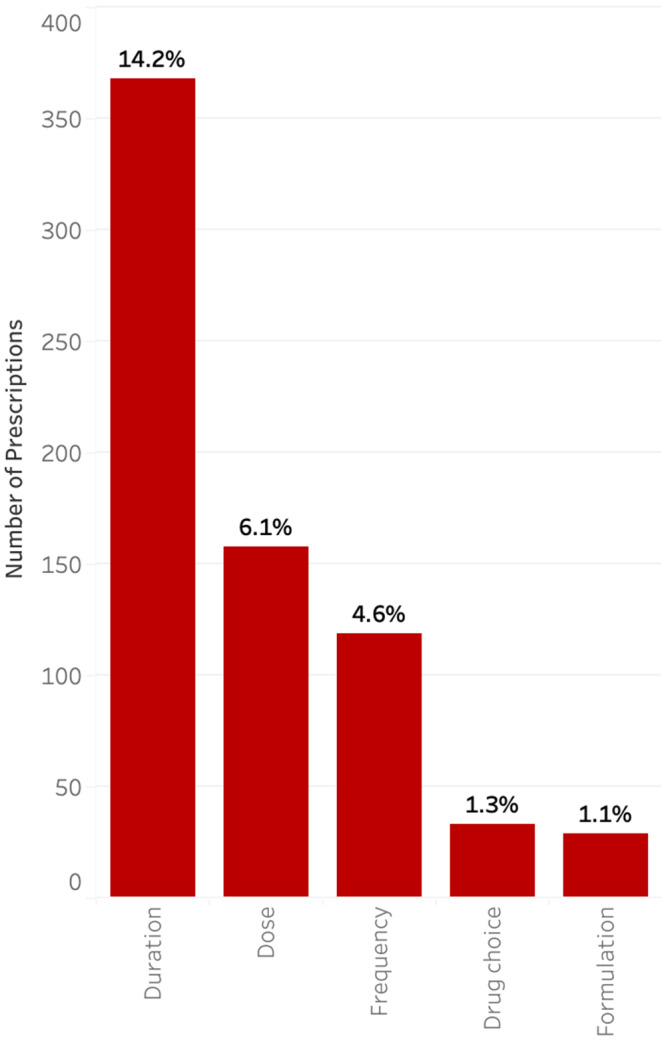
Rate of suboptimal prescriptions by type of discrepancy.

A total of 2,059 patients were discharged with an antimicrobial during the study period. The median age was 10 years (IQR 3–16), and median length of hospital stay was 3 days (IQR 1–7). Additional demographics are shown in Table 1. Most patients were discharged on only one antimicrobial (80.4%), with the rest discharged on two (13.1%), three (4.2%), or four or more (2.3%). A total of 2,593 prescriptions were included, of which 510 (19.7%) were suboptimal. The most common reason for classification as suboptimal was long duration (14.2%) followed by incorrect dose (6.1%) (Figure 1). Among prescriptions determined to be suboptimal for multiple reasons (33.2%), the most common reasons were duration (52.0%), followed by dose (22.3%).

**Table 1.** Demographic characteristics of study population

Characteristic	Value
Total patients, *n*	2059
Sex, no (%)	
Female	941 (45.7%)
Male	1118 (54.3%)
Age, median, y (IQR)	10 (3–16)
Insurance, *n* (%)	
Public insurance	1021 (49.6%)
Private insurance	1002 (48.7%)
Unknown	36 (1.7%)
Race/Ethnicity, *n* (%)	
Hispanic	846 (41.1%)
White or Caucasian	397 (19.3%)
Asian	300 (14.6%)
Black or African American	48 (2.3%)
Multiracial	38 (1.8%)
Native Hawaiian or Other Pacific Islander	29 (1.4%)
American Indian or Alaska Native	6 (0.3%)
Other	78 (3.8%)
Unknown	317 (15.4%)
Length of Stay, median day (IQR)	3 (1–7)
Number of Discharge Antimicrobials per patient, *n* (%)	
1	1655 (80.4%)
2	270 (13.1%)
3	87 (4.2%)
More than 3	47 (2.3%)

**Figure 2.**
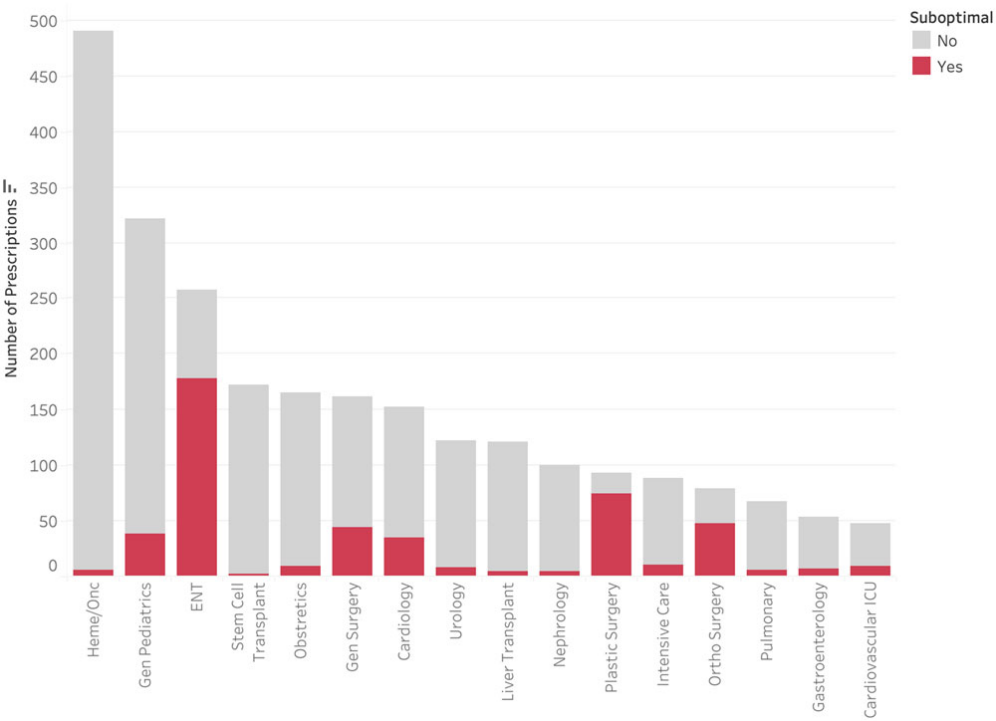
Rate of suboptimal prescriptions by medical service.

Prescriptions were additionally analyzed based on the prescribing medical service (Figure 2, Supplementary Table 1). The three highest prescribers of antimicrobials at discharge were hematology/oncology (490, 18.9%), followed by general pediatrics (322, 12.4%) and otolaryngology (ENT) (258, 9.9%). The services with the highest rates of prescriptions determined to be suboptimal were plastic surgery (80.6%), followed by ENT (69.0%) and orthopedic surgery (60.8%) (Figure 2).

**Figure 3.**
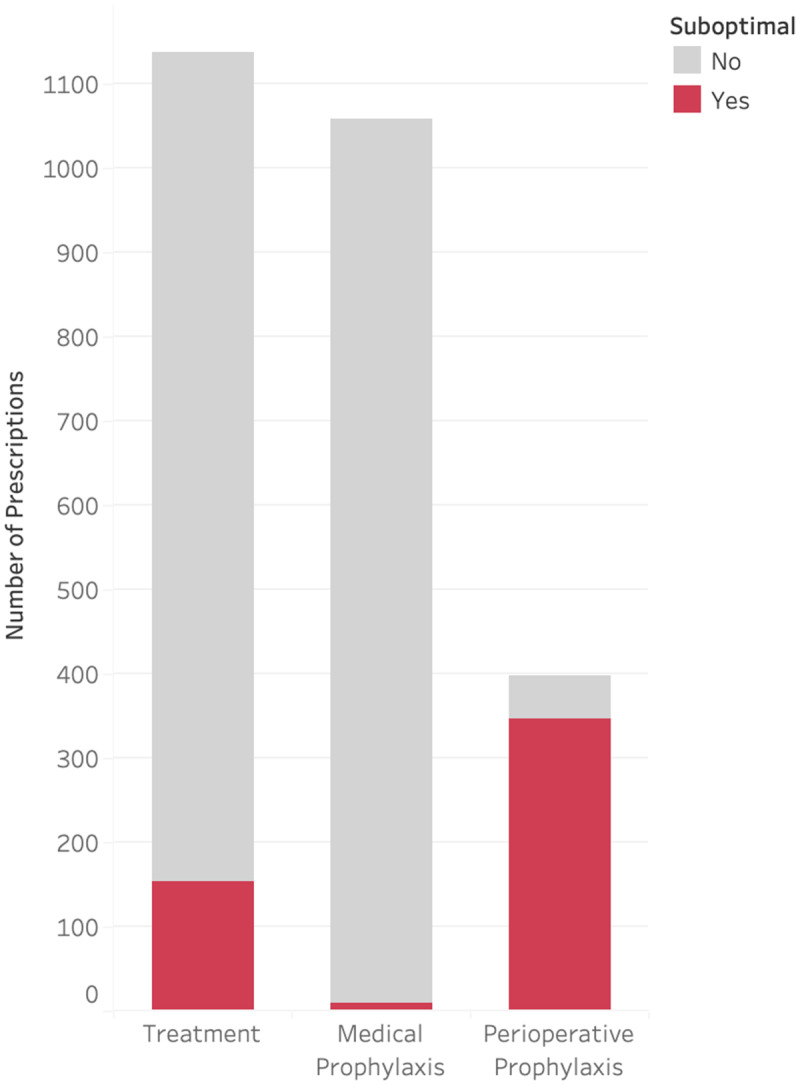
Rate of suboptimal prescriptions by indication.

**Figure 4.**
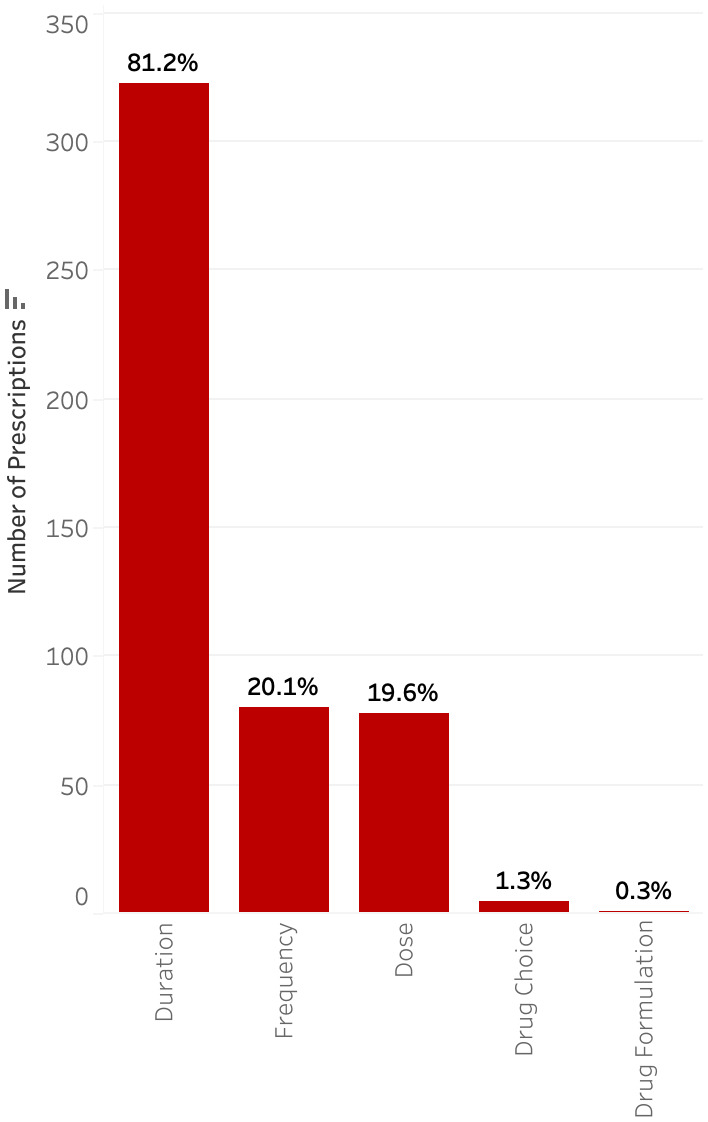
Type of discrepancy within suboptimal prescriptions for perioperative prophylaxis.

**Figure 5.**
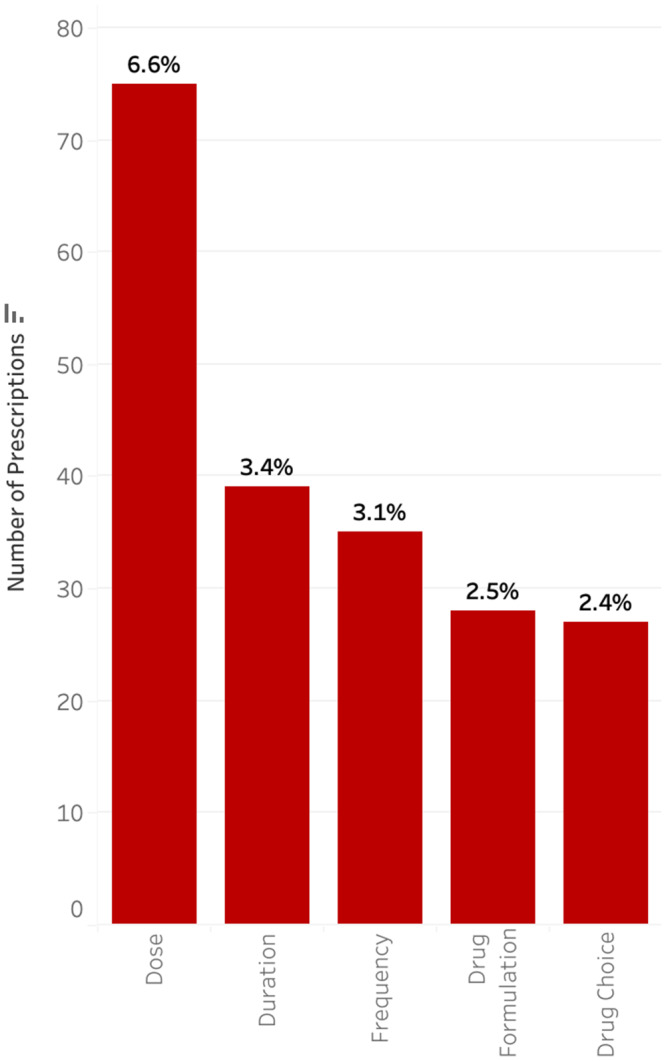
Type of discrepancy within suboptimal prescriptions for treatment of infections.

Only 1,137 (43.8%) of audited antimicrobials were prescribed for the treatment of an infection, with 1,058 (40.8%) for medical prophylaxis and 398 (15.3%) for perioperative prophylaxis (Figure 3). Less than 1% of medical prophylaxis prescriptions were suboptimal, whereas 87.2% of perioperative prophylaxis prescriptions were deemed to be suboptimal. The most common reasons for suboptimal perioperative prescriptions were duration (81.2%), followed by frequency (20.1%), and dose (19.6%) (Figure 4). Only 13.5% of prescriptions for treatment of an infection were suboptimal, primarily due to dose (6.6%), duration (3.4%), and frequency (3.1%) (Figure 5). The most common indications for antimicrobial treatment were urinary tract infection (UTI) (20.2%), followed by skin and soft tissue infection (SSTI) (14.5%) and appendicitis (10.8%). Rates of suboptimal discharge antimicrobial prescribing for treatment were highest for appendicitis (29.0%), followed by intra-abdominal process (17.6%), SSTI (15.2%), and community-acquired pneumonia (CAP) (13.6%) (Supplementary Figure 1). Reasons for treatment antimicrobial prescriptions to be classified as suboptimal differed by infection type, with drug choice being the most common reason for UTI (3.9%), dose for SSTI (6.7%) and appendicitis (22.5%), and duration for CAP (6.2%) (Supplementary Table 2).

**Figure 6.**
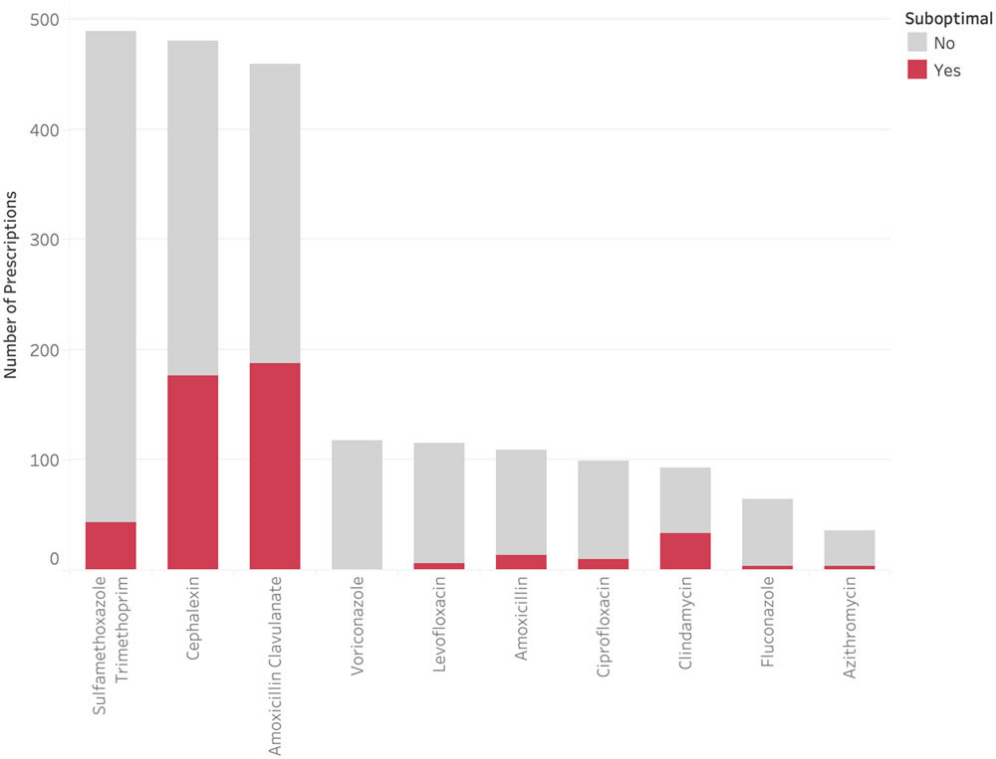
Rate of suboptimal prescriptions by antimicrobial.

Three antimicrobials (sulfamethoxazole-trimethoprim [SMX-TMP], amoxicillin-clavulanate, and cephalexin) made up the majority of discharge prescriptions (Figure 6). Sulfamethoxazole-trimethoprim had a comparatively low suboptimal rate at 9.0%, primarily due to long duration (8.6%). Amoxicillin-clavulanate and cephalexin had a high volume of discharge prescriptions and were frequently suboptimal (40.7% and 36.6%, respectively). Further breakdown of these antimicrobials by suboptimal classification can be found in Supplementary Table 3. Both amoxicillin-clavulanate and cephalexin were frequently prescribed for longer than recommended durations (30.8% and 29.5%, respectively) and suboptimal doses (16.0% and 11.4%, respectively). In addition, 6.1% of the ordered amoxicillin-clavulanate prescriptions had issues around formulation-dose mismatch. The most common issue with cephalexin orders was suboptimal dosing frequency (18.5%).

During ASP evaluation of the 510 suboptimal discharge prescriptions, 127 were found to be significant enough to raise concern about decreased efficacy or toxicity. Of these, the ASP reached out with recommendations to the provider on 54 (42.5%), while 73 (57.5%) of these prescriptions did not have recommendations made on them as the therapy was at or near completion prior to the ASP audit. Of these prescriptions where the ASP reached out to the provider with a recommendation to modify therapy, 46 (85.2%) resulted in a change accepted by the provider (Supplementary Table 4).

## Discussion

In this study, we found that 19.7% of antimicrobials prescribed at the time of hospital discharge were suboptimal. The rate of suboptimal discharge prescribing is similar to a prior study at a children’s hospital.^12^ Previous studies have shown that more than a third of hospitalized patients are given antibiotics after discharge.^8^ In this study, we found prolonged duration as the number one reason for suboptimal prescribing, which is consistent with the frequent use of excessive postoperative antibiotic duration seen in other work.^15^ Our study expands on this work and highlights that reasons for suboptimal discharge prescribing differ based on the antimicrobial prescribed, the indication for prescription, and the service prescribing the antimicrobial.

Of note, while 19.7% of discharge antimicrobials in this study were suboptimal, the rate differed significantly when divided between perioperative prophylaxis (87.2%), treatment (13.5%), and medical prophylaxis (0.9%). There are a number of explanations for these findings. First, in this study, medical prophylaxis was largely driven by hematology/oncology patients, whose care was heavily protocolized. In addition, the hematology/oncology service has at least one dedicated rounding pharmacist, giving greater opportunity for pharmacist input and feedback on prescriptions prior to patient discharge. Second, the majority of suboptimal perioperative prescriptions were due to excessive duration, likely reflecting differences in viewpoints between the ASP and surgeons on the appropriate length of perioperative antibiotic prescribing. The rate of suboptimal perioperative prescriptions we found was higher than in other studies, potentially reflecting different patient populations/mixes of surgical procedures.^16,17^ Although the optimal duration of antimicrobial prophylaxis is not known for every surgical procedure, the majority of procedures do not require prophylaxis beyond 24 hours of surgery, a position supported by a number of national guidelines.^18–20^ There is a growing body of literature suggesting potential harm to patients receiving unneeded days of perioperative prophylaxis, highlighting an area in need of quality improvement.^21^ Even if one does not consider duration, perioperative antimicrobials were found to be suboptimal in dose and dosing frequency approximately 20% of the time. Given these findings, we suggest that future discharge antimicrobial stewardship work focus on perioperative antibiotic prescribing.

Only 13.5% of antimicrobials indicated for the treatment of an infection were found to be suboptimal, with suboptimal dosing being the main area for improvement. A prior study of adult inpatients showed that UTI, SSTI, and CAP accounted for a large proportion of discharge antimicrobial prescriptions, and that nearly half of these were suboptimal.^22^ Adherence to standardized treatment pathways/algorithms may improve optimal discharge prescribing for the treatment of infection.^23,24^

Both cephalexin and amoxicillin-clavulanate prescriptions were suboptimal more than one-third of the time, mostly due to long durations. However, it is worth noting that amoxicillin-clavulanate prescriptions had issues with formulation-dosing mismatch 6.1% of the time. Amoxicillin-clavulanate has multiple formulations (e.g., amoxicillin: clavulanate ratios of 4:1, 7:1, 16:1), enabling different dosing strategies, based on the indication. Although this flexibility is important to avoid adverse effects caused by excessive clavulanate dosing, it presents a significant prescribing challenge.^25,26^ Given that higher oral clavulanate dosing may cause increased gastrointestinal side effects, this finding highlights an area for future stewardship interventions, such as electronic health record order sets that can guide prescribers to the appropriate formulation.

This study demonstrates that providers accept ASP recommendations of discharge antimicrobials more than 80% of the time. However, nearly two-thirds of suboptimal antimicrobial prescriptions did not result in an ASP recommendation owing to time between order and audit, demonstrating a limitation to this stewardship intervention. Future work should consider real-time interventions and increased clinical decision support to decrease the number of suboptimal prescriptions.

Strengths of this study include the large patient volume that captured all enteral discharge prescriptions over an entire calendar year. This large volume allowed us the ability to analyze the prescriptions by a number of clinically relevant subcategories. Another strength of this study was that each discharge antimicrobial was reviewed by the ASP within 72 hours, which allowed for the identification of indication instead of reliance on ICD-10 diagnosis codes, which may be less accurate. Of note, this study took place at a large, tertiary referral children’s hospital with a dedicated ASP performing daily audits. Other hospitals without these dedicated resources may not be able to implement similar practices or identify antimicrobial indications and suboptimal prescribing in the same manner. In addition, inhaled, injectable, and topical antimicrobials, as well as antimicrobials prescribed for noninfectious indications (e.g. amoxicillin-clavulanate for motility), were not evaluated. This study also did not evaluate the impact of suboptimal prescriptions on patient outcomes, which would help identify where ASP attention and resources must be focused.

In conclusion, we demonstrated that nearly 1 in 5 antimicrobial discharge prescriptions are suboptimal and that these rates can be substantially higher depending on the indication and medication. Stewarding of antimicrobials at hospital discharge is uniquely challenging as these prescriptions occur during transitions of care and changes to the treatment plan after patient discharge and prescription pick-up from the pharmacy are less likely to occur. Future work should investigate ways to lower rates of suboptimal antimicrobial prescribing. Perioperative discharge antibiotics have a particularly high rate of suboptimal prescriptions and deserve increased attention.

## Supporting information

Zhang et al. supplementary material Zhang et al. supplementary material

## Supplementary material

For supplementary material accompanying this paper visit http://doi.org/10.1017/ash.2023.488. click here to view supplementary material
