# Supplementary material for: Suboptimal antimicrobial discharge prescriptions at a tertiary referral children’s hospital
Source: Antimicrob Steward Healthc Epidemiol. 2023 Dec 4;3(1):e223. doi: 10.1017/ash.2023.488 (PMC10753499; doi:10.1017/ash.2023.488)

**Supplemental Material**

**Supplementary table 1. Discharge prescriptions by service**

| Discharge Service | Number (%) |
| --- | --- |
| Hematology/Oncology | 490 (18.9%) |
| General Pediatrics | 322 (12.4%) |
| Otolaryngology (ENT) | 258 (9.9%) |
| Stem Cell Transplant | 172 (6.6%) |
| Obstetrics | 166(6.4%) |
| General Surgery | 162 (6.2%) |
| Cardiology | 153 (5.9%) |
| Urology | 122 (4.7%) |
| Liver Transplant | 121 (4.7%) |
| Nephrology | 100 (3.9%) |
| Plastic Surgery | 93 (3.6%) |
| Intensive Care | 89 (3.4%) |
| Ortho Surgery | 79 (3.0%) |
| Pulmonary | 68 (2.6%) |
| Gastroenterology | 54 (2.1%) |
| Cardiovascular ICU | 48 (1.9%) |

**Supplementary table 2. Suboptimal prescription classification by infection type**

| UTI (number, %) | | | | | |
| --- | --- | --- | --- | --- | --- |
|  | Duration | Dose | Drug Choice | Drug Formulation | Frequency |
| Optimal | 228 (99.1%) | 225 (97.8%) | 221 (96.1%) | 230 (100.0%) | 223 (97.0%) |
| Suboptimal, Long/high | 2 (0.9%) | 1 (0.4%) | 9 (3.9%) |  | 7 (3.0%) |
| Suboptimal, short/low |  | 4 (1.7%) |  |  |  |
| SSTI (number, %) | | | | | |
|  | Duration | Dose | Drug Choice | Drug Formulation | Frequency |
| Optimal | 156 (94.6%) | 154 (93.3%) | 162 (98.2%) | 165 (100.0%) | 157 (95.2%) |
| Suboptimal, Long/high | 9 (5.5%) | 2 (1.2%) | 3 (1.8%) |  | 1 (0.6%) |
| Suboptimal, short/low |  | 9 (5.5%) |  |  | 7 (4.2%) |
| Appendicitis (number, %) | | | | | |
|  | Duration | Dose | Drug Choice | Drug Formulation | Frequency |
| Optimal | 120 (96.8%) | 96 (77.4%) | 117 (94.3%) | 111 (89.5%) | 119 (96.0%) |
| Suboptimal, Long/high | 3 (2.4%) | 10 (8.0%) | 7 (5.7%) | 13 (10.5%) | 2 (1.6%) |
| Suboptimal, short/low | 1 (0.8%) | 18 (14.5%) |  |  | 3 (2.4%) |
| CAP (number, %) | | | | | |
|  | Duration | Dose | Drug Choice | Drug Formulation | Frequency |
| Optimal | 76 (93.8%) | 80 (98.8%) | 80 (98.8%) | 79 (97.5%) | 79 (97.6%) |
| Suboptimal, Long/high | 4 (4.9%) |  | 1 (1.2%) | 2 (2.5%) | 1 (1.2%) |
| Suboptimal, short/low | 1 (1.2%) | 1 (1.2%) |  |  | 1 (1.2%) |

**Supplementary table 3. Suboptimal prescription classification by antimicrobial**

| Clindamycin (number, %) | | | | | |
| --- | --- | --- | --- | --- | --- |
|  | Duration | Dose | Drug Choice | Drug Formulation | Frequency |
| Optimal | 71 (76.3%) | 79 (85.0%) | 87 (93.5%) | 93 (100.0%) | 91 (97.9%) |
| Suboptimal, Long/high | 22 (23.7%) | 14 (15.0%) | 6 (6.5%) |  | 1 (1.1%) |
| Suboptimal, short/low |  |  |  |  | 1 (1.1%) |
| Amoxicillin/Clavulanate Potassium (number, %) | | | | | |
|  | Duration | Dose | Drug Choice | Drug Formulation | Frequency |
| Optimal | 327 (69.0%) | 398 (84.0%) | 471 (99.4%) | 445 (93.9%) | 463 (97.7%) |
| Suboptimal, Long/high | 146 (30.8%) | 14 (2.9%) | 3 (0.6%) | 29 (6.1%) | 4 (0.8%) |
| Suboptimal, short/low | 1 (0.2%) | 62 (13.1%) |  |  | 7 (1.5%) |
| Cephalexin (number, %) | | | | | |
|  | Duration | Dose | Drug Choice | Drug Formulation | Frequency |
| Optimal | 338 (70.3%) | 426 (88.6%) | 478 (99.4%) | 481 (100.0%) | 392 (81.5%) |
| Suboptimal, Long/high | 142 (29.5%) | 7 (1.4%) | 3 (0.6%) |  | 1 (0.2%) |
| Suboptimal, short/low | 1 (0.2%) | 48 (10.0%) |  |  | 88 (18.3%) |
| Sulfamethoxazole-Trimethoprim (number, %) | | | | | |
|  | Duration | Dose | Drug Choice | Drug Formulation | Frequency |
| Optimal | 445 (91.4%) | 485 (99.6%) | 487 (100.0%) | 487 (100.0%) | 485 (99.6%) |
| Suboptimal, Long/high | 42 (8.6%) |  |  |  | 2 (0.4%) |
| Suboptimal, short/low |  | 2 (0.4%) |  |  |  |

**Supplementary table 4. Modifications on Discharge prescriptions**

| Modifications | Number (%) |
| --- | --- |
| Increase dose | 26 (48.1%) |
| Decrease dose | 5 (9.3%) |
| Increase Frequency | 4 (7.4%) |
| Decrease frequency | 1 (1.9%) |
| Length duration | 2 (3.7%) |
| Shorten duration | 1 (1.9%) |
| Clarify indication/plan | 1 (1.9%) |
| Stop treatment | 2 (3.7%) |
| Other | 2 (3.7%) |
| Change (C&S)* | 6 (11.1%) |
| Change (availability)* | 3 (5.6%) |
| Change (ADR)* | 1 (1.9%) |

*Change (C&S): Recommend change based on available culture and susceptibility results.

*Change (availability): Recommend change due to medication not being available.

*Change (ADR): Recommend change due to suspected or increased risk of drug adverse reaction.

**Supplementary figure 1. Rate of suboptimal prescriptions by treatment indication**


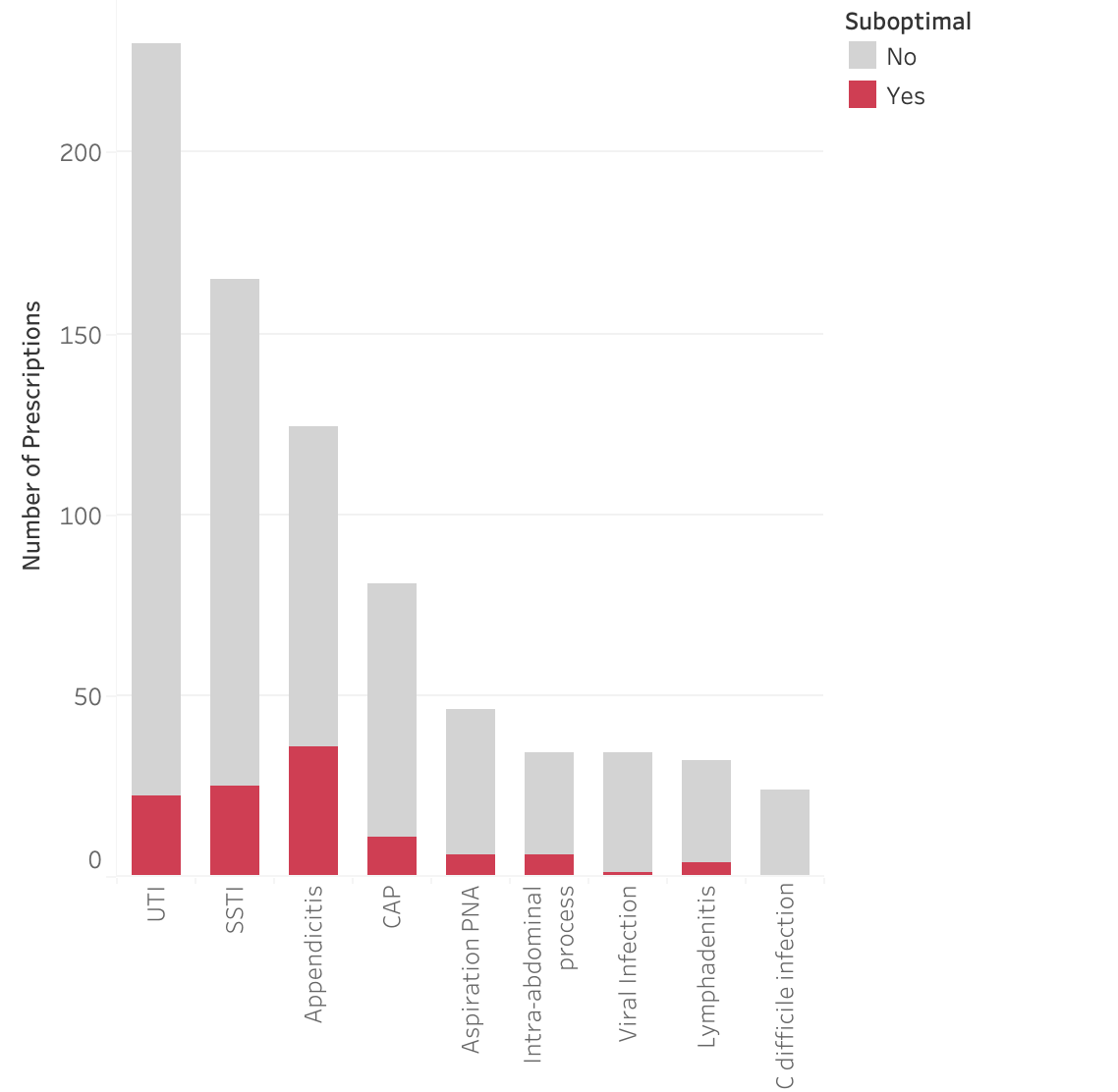

Supplement: Zhang et al. supplementary material [file S2732494X23004886sup001.docx]
